# Expression of Nectin-4 in Variant Histologies of Bladder Cancer and Its Prognostic Value—Need for Biomarker Testing in High-Risk Patients?

**DOI:** 10.3390/cancers14184411

**Published:** 2022-09-11

**Authors:** Severin Rodler, Lennert Eismann, Boris Schlenker, Jozefina Casuscelli, Isabel Brinkmann, Andrea Sendelhofert, Raphaela Waidelich, Alexander Buchner, Christian Stief, Gerald Bastian Schulz, Stephan Ledderose

**Affiliations:** 1 Department of Urology, University Hospital of Munich, 81377 Munich, Germany; 2 Comprehensive Cancer Center, University Hospital of Munich, 81377 Munich, Germany; 3 Department of Pathology, Ludwig Maximilian University Munich, 81377 Munich, Germany

**Keywords:** Nectin-4, squamous cell carcinoma, neoadjuvant therapy, adjuvant therapy, antibody–drug conjugate

## Abstract

**Simple Summary:**

Variant histologies of bladder cancer present at advanced stage and are often treated with radical cystectomy. As Nectin-4 appears to be a promising target for novel therapies in conventional bladder cancer, we aimed to analyze the expression of Nectin-4 and its prognostic value in variant histologies of bladder cancer. We found a high expression of Nectin-4 in squamous cell carcinoma and adenocarcinoma and a low expression in sarcomatoid urothelial carcinoma. No impact of Nectin-4 expression on survival has been detected in our study. Our study reveals the need to perform further biomarker testing for Nectin-4 in prospective trials including patients with variant histologies.

**Abstract:**

Variant histologies of bladder cancer (BC) often present with advanced tumor stage and the status of perioperative therapy is unclear. Thereby, squamous cell carcinoma (SCC), adenocarcinoma (ADENO), and sarcomatoid urothelial carcinoma (SARCO) are the most frequent variants. Nectin-4 has emerged as a highly interesting target in BC and might guide therapeutic application of antibody–drug conjugates (ADC). We therefore aimed to investigate expression patterns and prognostic value of Nectin-4 in variant histologies of BC. A single-center retrospective analysis was conducted of patients who underwent radical cystectomy (RC) for BC and revealed variant histologies of BC in the final specimens. Immunohistochemical staining for Nectin-4 was performed on tissue microarrays with 59 SCC, 22 ADENO, and 24 SARCO, and Nectin-4 expression was scored using the histochemical scoring system (H-score). Overall survival (OS) and progression-free survival (PFS) was calculated by Kaplan–Meier method. Median expression of Nectin-4 was 150 (range 0–250) in SCC, 140.5 (range 30–275) in ADENO, and 10 (0–185) in SARCO, with significantly lower levels for SARCO compared to SCC or ADENO (*p* < 0.001). For SCC, ADENO or SARCO no differences regarding OS or PFS were observed based on Nectin-4 expression levels (*p* > 0.05). Multivariate analysis revealed nodal stage as an independent prognostic factor for OS and PFS and metastases for PFS but not Nectin-4 expression. In conclusion, Nectin-4 was not prognostic in histological subtypes of BC in our study cohort. However, the high expression of Nectin-4 in SCC and ADENO might guide future treatment with novel Nectin-4-directed ADCs and provide this high-risk patient collective with a new promising therapeutic option. Testing Nectin-4 expression as a biomarker should be considered in trials with SARCO, where low Nectin-4 expression has been observed.

## 1. Introduction

Nectin cell adhesion molecule 4 (Nectin-4) is a highly innovative and promising treatment target in urothelial carcinoma of the bladder (UC) [1]. Nectin-4 is a single-pass type I immunoglobulin-like membrane protein and it mediates various cell functions such as proliferation, differentiation, migration, and invasion [2]. The protein is physiologically expressed on tissues such as skin, bladder, and lung at weak to moderate levels. Interestingly, Nectin-4 is overexpressed in many types of cancers, including UC [3]. Consequently, enfortumab vedotin, an antibody–drug conjugate (ADC) that targets Nectin-4, has significantly improved survival in patients with locally advanced or metastatic urothelial carcinoma [4]. As the ADC directly targets the Nectin-4 molecule, the Nectin-4 expression on cancer cells is relevant for the efficacy of this class of drugs [5]. While Nectin-4 expression is consistently very high in UC, there are fewer data on expression of Nectin-4 in variant histologies of BC [3,4,6,7].

Variant histologies account for 25% of all bladder cancers (BC) in Western countries [6]. They can be classified based on the presence of urothelial cells into urothelial and non-urothelial variants. Thereby, pure squamous cell carcinoma (SCC) and adenocarcinoma of the bladder (ADENO) represent two frequent non-urothelial variants, and sarcomatoid urothelial carcinoma (SARCO) an urothelial variant [7]. Patients revealing those subtypes tend to present with advanced disease at the time of first diagnosis compared to conventional UC, and the prognosis is consequently poor [8]. Therefore, new treatment options for those patients become increasingly important to improve outcomes of those patients at high risk. However, patients with variant histologic subtypes of BC used to be underrepresented or excluded from most clinical trials evaluating the role of systemic therapies, including those targeting Nectin-4 [4,9]. Nectin-4 shows a heterogenous expression in variant histologies with low expression in SARCO and small cell [10]. However, mainly small cohorts are described [11]. In addition, little is known about the prognostic role of Nectin-4 in variant histologies of BC, which could serve as a basis for ADC therapy.

Therefore, we aimed to investigate the expression pattern of Nectin-4 in pure SCC, pure ADENO, and SARCO in patients at the time of radical cystectomy (RC). Further, we analyzed the prognostic potential of Nectin-4 expression in those three variants of BC.

## 2. Materials and Methods

### 2.1. Study Setting and Patients

We retrospectively selected patients with pure SCC (*n* = 61), pure ADENO (*n* = 23), and SARCO (*n* = 26) who underwent RC at the Department of Urology of LMU Munich between 2005 and 2021. Preoperative imaging was performed to exclude metastatic disease. Patients then underwent radical cystectomy (RC) in a standardized procedure, including lymphadenectomy and urinary diversion with either ileal neobladder or ileal conduit. All procedures were performed by trained urologists. Histopathological analysis was performed by experienced pathologists at the Department of Pathology, LMU Munich. The possibility of a secondary tumor manifestation by direct invasion or hematogenous seeding was excluded by careful morphologic and, if necessary, immunohistochemical examination of the surgical specimens, taking into account clinical data (especially a history of prior cancer) as well as radiologic and endoscopic findings in accordance with WHO recommendations. All available histologic specimens were reviewed to confirm tumor type and stage. Staging was performed according to the AJCC/UICC TNM staging guidelines (8th edition) and only tumors that met the criteria for pure SCC, pure ADENO, and SARCO according to the latest WHO classification of genitourinary tumors were included.

Formalin-fixed and paraffin-embedded (FFPE) tumor tissue blocks were used for construction of tissue microarrays (TMA). In two patients with SCC, one patient with ADENO, and two patients with SARCO, no representative tumor material was available, and the corresponding patients were excluded from further analysis.

### 2.2. Follow-Up

Patients undergoing RC at our center are prospectively included into an institutional database. They are followed-up according to the EAU guideline either at our highly specialized outpatient clinic or externally by resident urologists [12]. In addition, all patients are followed-up at least once per year via mail, as described [13].

### 2.3. Tissue Microarray Construction

Nectin-4 expression was examined by immunohistochemical staining of TMAs containing tumor cores from 59 patients with SCC, 22 patients with ADENO, and 24 with SARCO. TMAs were generated with three 1 mm tumor cores from different tumor areas of representative FFPE samples. Triplicates of each sample were used to minimize tissue loss and to overcome tumor heterogeneity.

### 2.4. Immunohistochemistry

Immunohistochemistry analyses were performed using Anti-PVRL-4 (Nectin-4) rabbit antiserum (HPA010775; atlas antibodies, Lund, Sweden) diluted 1:100. TMA sections were subjected to heat retrieval with pH 8.0 (Epitope Retrieval Solution, Leica Microsystems, Wetzlar, Germany). After incubation with the primary antibody for 60 min at room temperature, bound antibodies were detected by the use of MACH 3 Rabbit HRP Polymer system (Biocare Medical, Pacheco, CA, USA). Reaction was visualized with DAB+ (Agilent Technologies, Santa Clara, CA, USA). Sections were then counterstained in hematoxylin, dehydrated, and mounted.

### 2.5. Semiquantitative Analysis of Nectin-4 Expression

Immunoreactivity was scored using the histochemical scoring system (H-score) as previously described [14]. The H-score incorporates both the membranous and cytoplasmatic staining intensity and the percentage of stained cells at each intensity level. Intensity was scored as 0 (no evidence of staining), 1 (weak staining), 2 (moderate staining), and 3 (strong staining). The final H-score is the sum of the intensity values multiplied by the percentage of stained cells. The mean of readings in three tumor cores per patient was calculated and represented the final H-score in each tumor sample. If one TMA core was lost or contained no tumor tissue, this core was excluded for overall score. Specimens were then classified as low (H-score < 100), intermediate (H-score 100–200), and high (H-score > 200).

### 2.6. Statistical Analysis

Statistical analysis was performed by chi-square test, Kruskal–Wallis analysis, or Mann–Whitney U-test for group comparisons. Overall survival (OS) and progression-free survival (PFS) were calculated by the Kaplan–Meier method. Survival differences were calculated by log-rank test. Multivariate analysis was performed using Cox regression model adjusting for age, gender, and TNM stage. A *p* value less than 0.05 was considered statistically significant. All calculations were performed using Prism 9 software (Graphpad Software, San Diego, CA, USA).

## 3. Results

We analyzed patient characteristics of the patient cohort with SCC, ADENO, and SARCO, immunoreactivity of Nectin-4, and impact of Nectin-4 expression on survival in the respective variant histology of BC.

### 3.1. Patients

A total of 1600 patients underwent RC at our academic center between 2005 and 2021. Archived tissue samples from 59 patients with SCC of the bladder, 22 patients with ADENO, and 24 patients with SARCO were selected from our prospectively conducted database, and the expression of Nectin-4 was evaluated by immunohistochemistry on TMAs.

Median follow-up of the study cohort was 14 (range 0–175) months. Median age of all patients was 67 (range 37–89) years. A total of 56 patients were male and 49 female. There was no difference in age (*p* = 0.497) or gender (*p* = 0.267) across the three histological subtypes.

In the cohort of SCC, 8 patients revealed pT2, 34 patients pT3, and 15 patients pT4 in the final histological specimens. In ADENO, 1 patient presented with pT2, 14 with pT3, and 6 with pT4. In SARCO, 1 presented with pT2, 18 with pT3, and 5 patients with pT4. Further patient characteristics are listed in Table 1.

### 3.2. Expression of Nectin-4 in SCC, ADENO, and SARCO

**Figure 1:**
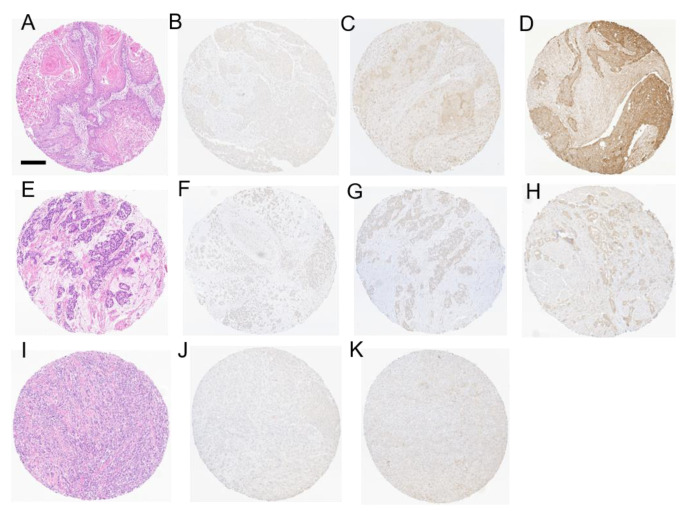
Variant histologies of bladder cancer and Nectin-4 expression levels. (**A**–**D**) show representative photomicrographs of tissue microarray (TMA) samples with SCC (**A**, H&E) and weak (**B**), intermediate (**C**), and high Nectin-4 expression (**D**). (**E**–**H**) depict representative photomicrographs of TMA samples with ADENO (**E**, H&E) and weak (**F**), intermediate (**G**), and high Nectin-4 expression (**H**). (**I**–**K**) show representative photomicrographs of TMA samples with SARCO (**I**, H&E) and weak (**J**) and intermediate (**K**) Nectin-4 expression. In SARCO, there was no high Nectin-4 expression detected. H&E, hematoxylin–eosin; SCC, pure squamous cell carcinoma of the bladder; ADENO, adenocarcinoma of the bladder; SARCO, sarcomatoid urothelial carcinoma of the bladder; scale bar 200 µm.

First, we examined the expression of Nectin-4 by immunohistochemistry in SCC, ADENO, and SARCO. As described in the literature, a partly cytoplasmic, partly membranous staining for Nectin-4 was observed in tumor cells [15,16]. Immunoreactivity was scored using the H-score as described. Patients were then stratified as depicted into Nectin-4^low^, Nectin-4^intermediate^, and Nectin-4^high^ based on expression of Nectin-4 (Figure 1, Supplementary Figure S1).

**Figure 2:**
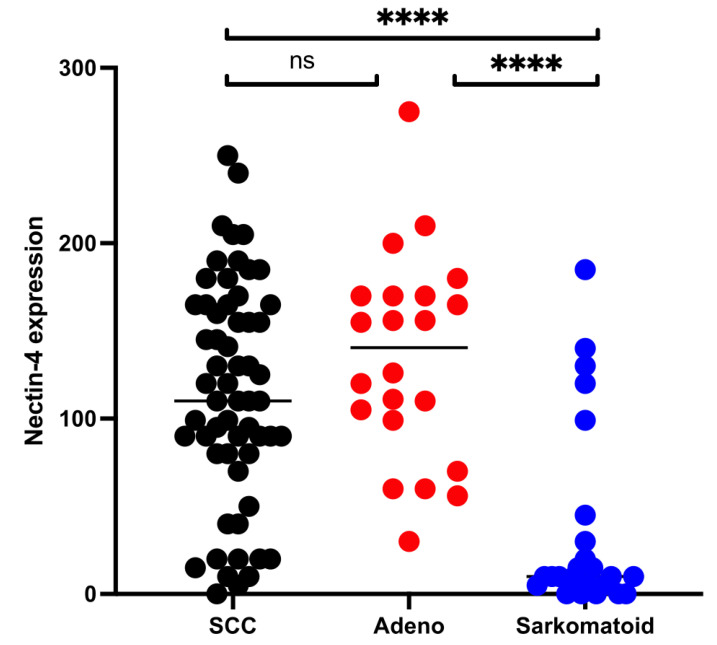
Nectin-4 expression (H-score) in different variant histologies of bladder cancer. Immunohistochemical expression of Nectin-4 for patients with pure squamous cell carcinoma (SCC—black), adenocarcinoma (ADENO—red), and sarcomatoid urothelcarcinoma (SARCO—blue) was evaluated by histochemical scoring system (H-score). Group comparisons were performed by Mann–Whitney U-test; ns: not significant, ****: *p* < 0.001.

Median expression of Nectin-4 was 150 (range 0–265) in SCC, 140.5 (range 30–270) in ADENO, and 10 (0–165) in SARCO. There was no statistical difference in the expression levels of Nectin-4 in SCC and ADENO (*p* = 0.2369). An H-score of 15 or less was observed in five patients (8.5%) with SCC and in no patient with ADENO. Interestingly, SARCO revealed a significantly lower expression of Nectin-4 compared to SCC (*p* < 0.001) and ADENO (*p* < 0.001). Thus, in SARCO, 16 patients (66.7%) revealed an H-score of 15 or below (Figure 2).

### 3.3. Nectin-4-Dependent Survival

Next, we analyzed OS and PFS for our patient cohort depending on the expression of Nectin-4. Nectin-4 expression was classified into low, intermediate, and high, as described. The median OS of the study cohort was 17 (2–92) months and median PFS was 14 (1–44) months. For SCC, the median OS for Nectin-4^high^ patients was 7 months and did not significantly differ from Nectin-4^intermediate^ (median OS 10 months) and Nectin-4^low^ patients (median OS: not reached; *p* = 0.246). PFS was not significantly different for the described subgroups (median PFS Nectin-4^high^: 11.5, Nectin-4^intermediate^: 10, and Nectin-4^low^ patients not reached, *p* = 0.361). For ADENO, OS (*p* = 0.786) and PFS (*p* = 0.442) were not significantly different based on Nectin-4 expression when categorized into the three expression groups with a median OS of 48, 93, and 40 months and median PFS of 45, 9, and 39 months for Nectin-4^low^, Nectin-4^intermediate^, and Nectin-4^high^ patients, respectively.

**Figure 3:**
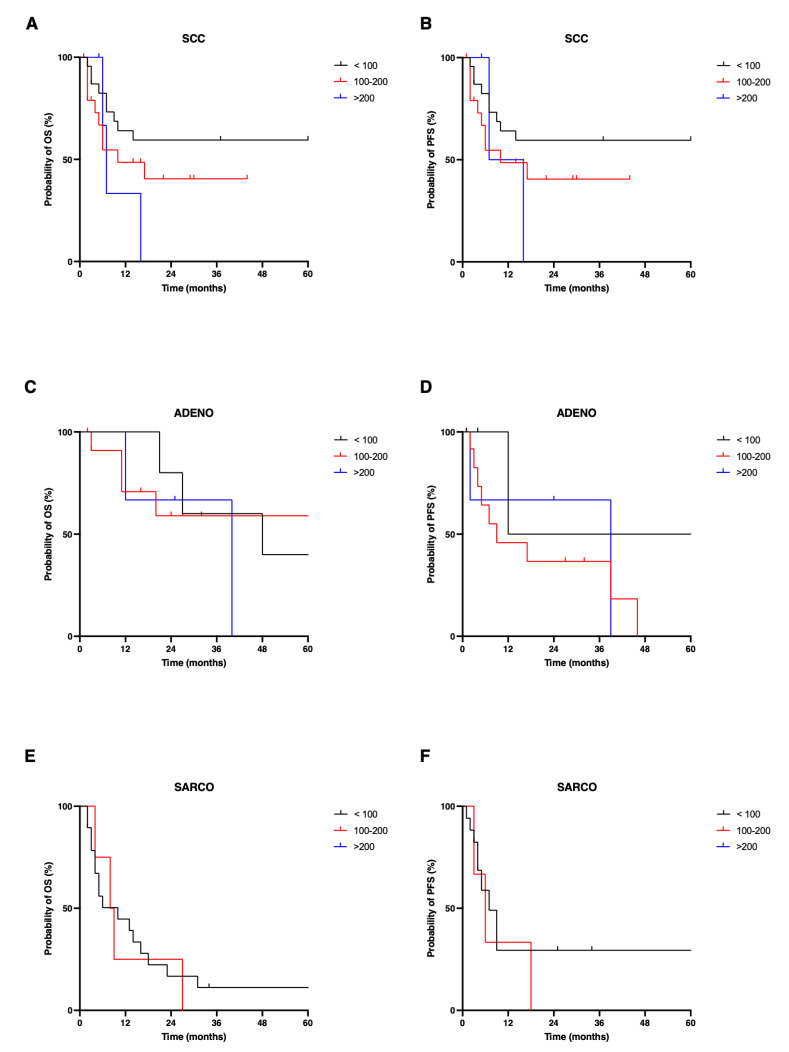
Overall (OS) and progression-free survival (PFS) in variant histologies of bladder cancer. Expression of Nectin-4 was classified as low (H-score < 100, black), intermediate (H-score 100–200, red), and high (H-score > 200, blue). Group comparisons were performed by log-rank test. OS (**A**) and PFS (**B**) of squamous cell carcinoma (SCC), OS (**C**) and PFS (**D**) of adenocarcinoma (ADENO) and OS (**E**) and PFS (**F**) of sarcomatoid urothelicarcinoma (SARCO) were analyzed. In SARCO, there are no patients with a H-score of >200. OS, overall survival; PFS, progression-free survival; SCC, pure squamous cell carcinoma of the bladder; ADENO, adenocarcinoma of the bladder; SARCO, sarcomatoid urothelial carcinoma of the bladder.

As none of the SARCO tissues revealed high expression of Nectin-4, only the intermediate and low group were compared. Here, OS within the Nectin-4^low^ group (median: 10 months) and Nectin-4^intermediate^ group (median: 8.5 months) showed no significant difference (*p* = 0.798). Further, there was no difference in PFS, with a median of 7 months in the Nectin-4^low^ group and 6 months in the Nectin-4^intermediate^ (*p* = 0.495, Figure 3).

### 3.4. Multivariate Analysis

**Figure 4:**
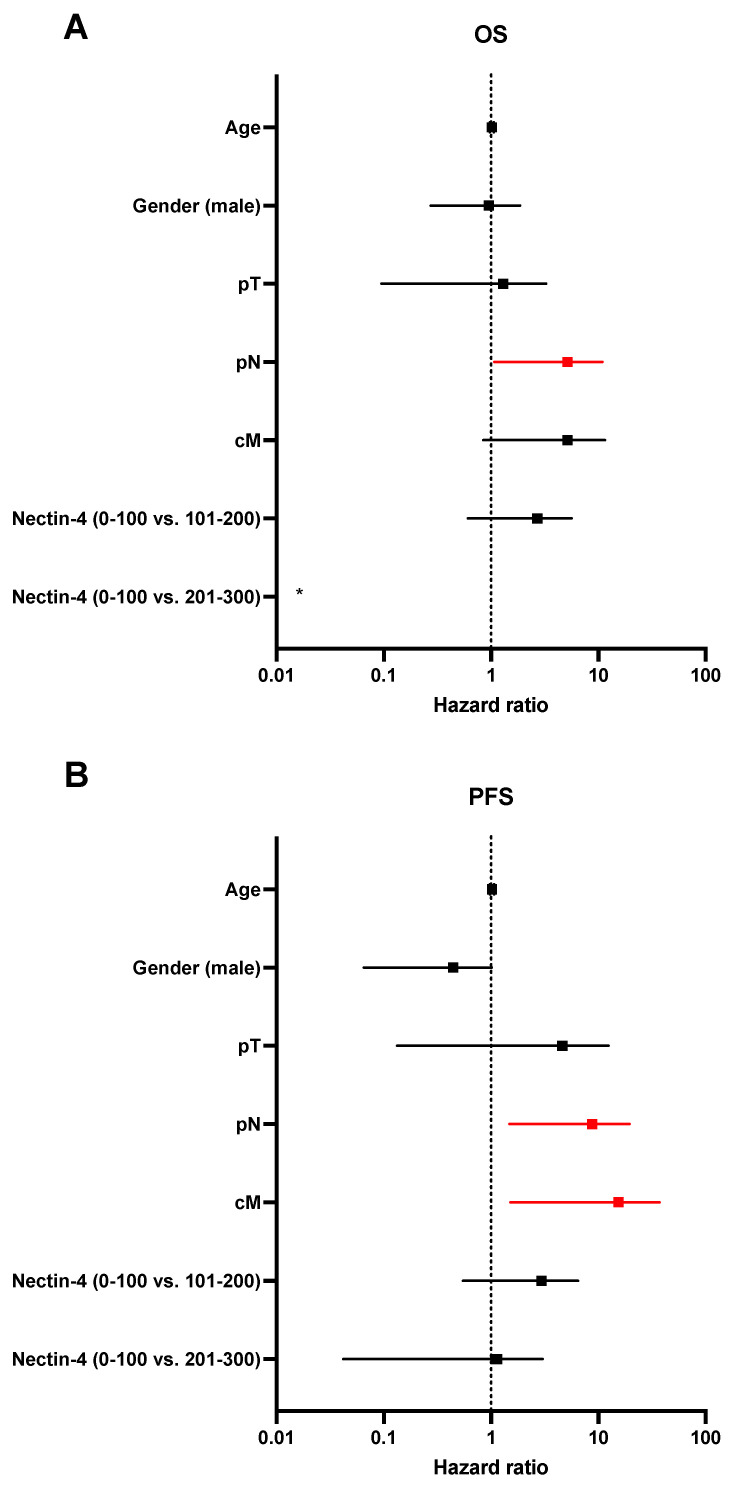
Multivariate analysis for OS and PFS in SCC. Age, gender, T-stage (pT), nodal stage (pN), radiological detected metastases (cM), and Nectin-4 expression were used as variables and independent prognostic potential evaluated for OS (**A**) and PFS (**B**). *p* values less than 0.05 were considered as statistically significant and groups are marked in red. * Data are outside of the axis limit due to sample size (HR: 0, 95% CI 1.221 × 10^−188^ to 1.182 × 10^177^).

Finally, we performed multivariate analysis including age, gender, T-stage, nodal stage, radiological metastases, and Nectin-4 expression. Nodal stage is independently prognostic for OS (HR: 3.43, 95% CI 1.08–10.92) and PFS (HR: 5.38, 95% CI 1.48–19.53) in SCC and metastases for PFS (HR: 7.52, 95% CI 1.52–37.18). Multivariate analysis for ADENO and SARCO was not performed due to the limited number of patients (Figure 4).

## 4. Discussion

This is one of the most comprehensive analyses of Nectin-4 expression in variant histologies of BC and its prognostic impact on survival. While previous studies have already revealed expression patterns in urothelial carcinoma and variant histologies [11], we further provide an analysis of the impact on survival. This study reveals a high Nectin-4 expression in pure SCC and pure ADENO and low Nectin-4 expression in SARCO. Nectin-4 expression is not prognostic for PFS and OS in our cohort.

While Nectin-4 expression is generally low in normal tissues, it is increased in several tumor types and is associated with tumor progression in breast, urothelial, pancreatic, lung, and ovarian cancers [3]. In pancreatic cancer, Nectin-4 expression is correlated with poor outcome [17], and in UTUC, high expression of Nectin-4 is associated with poor prognosis in high-risk patients [16]. In our cohort of patients at high risk of tumor progression and with variant histologies of BC, no correlation between Nectin-4 expression and PFS or OS was observed.

Nectin-4 has been demonstrated to be generally highly expressed in UC and, consequently, trials exploring enfortumab vedotin in UC do not use Nectin-4 expression as a stratifying factor [4]. For example, in metastatic UC, high expression of Nectin-4 was observed with an H-score of 290 (range: 14 to 300) [18]. In luminal and basal subtypes of UC, Nectin-4 expression was necessary for sensitivity of enfortumab vedotin, and downregulation of Nectin-4 led to ADC resistance in vitro [19]. Comparable data are highly limited for SCC and ADENO, which are considered as non-urothelial variants of BC and cannot be classified as luminal or basal subtypes [7]. Consequently, current clinical management of variant histologies of BC is mostly analogous to pure UC with lacking evidence of treatment efficacy [20]. There is an urgent need for deeper insights into Nectin-4 expression and the efficacy of enfortumab vedotin in variant histologies of BC.

Several variant histologies of BC, namely, SCC, ADENO, and small cell neuroendocrine carcinoma, have been shown to express Nectin-4 [14], but those early studies enclosed only small case numbers [11]. In our comparatively large cohort, SCC and ADENO revealed high expression levels for Nectin-4. Despite the histological difference between pure UC and non-urothelial variants such as SCC and ADENO, we report high expression level of Nectin-4 in those rare tumor subtypes. Therefore, treatment of advanced or metastatic non-urothelial variants SCC and ADENO with Nectin-4 targeting ADCs might be a valid option.

In contrast, SARCO revealed low expression levels, with 66.7% of all patients showing an H-score of 15 or below. SARCO is a rare histologic subtype of urothelial carcinoma, where areas that are histomorphologically indistinguishable from sarcomas are adjacent to a conventional urothelial component. Previous studies suggest that the poor prognosis of SARCO is due to the sarcomatoid component as it represents the end stage of urothelial carcinoma dedifferentiation [21,22]. Consequently, one aim of our study was to test whether the sarcomatoid component could be a target for anti-Nectin-4-directed therapies. However, Nectin-4 expression in the conventional urothelial component of SARCO might also be different from that in conventional UC and should therefore be investigated in further studies. With regard to molecular characterization, SARCO is classified as a basal subtype [23]. As reported by Chu et al., basal subtypes in UC are associated with lower expression levels of Nectin-4 [19]. Further, Hoffman-Censits et al. revealed a low expression rate of only 10% Nectin-4 positive tumors in a cohort of 10 patients with SARCO [11]. Correspondingly, our results are in line with the current literature and disclose the character of the rare SARCO. As already mentioned, the efficacy of ADC directed against Nectin-4 depends on Nectin-4 expression in both basal and luminal subtypes [19]. Our data show markedly reduced Nectin-4 expression in SARCO. Nevertheless, some patients showed moderate Nectin-4 expression. Future clinical trials evaluating Nectin-4 targeting agents should include patients with SARCO upon immunohistochemical Nectin-4 expression staining, to adequately evaluate response to therapy.

Based on our data, it seems that there is a heterogeneity in Nectin-4 expression across different histologic subtypes of BC. Variant histologies of BC often present at advanced stages at the time of RC [7], and systemic treatment options are limited by exclusion of these variants from clinical studies. To date, we are witnessing the advent of ADCs as potential standard treatment in advanced and metastatic bladder cancer [24]. Immunohistochemical stain of ADC targets, such as Nectin-4, Trop-2, or HER-2 [25], should be promoted to allow treatment allocation also in variant histologies. While we defined relevant Nectin-4 expression levels in pure SCC and pure ADENO, potentially warranting therapy with enfortumab vedotin in these tumors, further studies of targets in variant histologies are urgently needed. Nevertheless, the clinical impact of the expression level of Nectin-4 and other targets should be the focus for future clinical trials. Correlation of expression level and response to ADC targets are crucial components for stratifying patients for individualized therapy.

The outcome of patients depending on Nectin-4 expression has not been described in prospective trials up to date [26]. Our study reveals no statistically significant difference in survival depending on expression of Nectin-4. Interestingly, in SCC a clear tendency towards worse outcomes is observed across the three expression levels. Patients with more aggressive disease might have a higher Nectin-4 expression and are therefore a subgroup that particularly needs to be looked at in prospective studies.

The study is limited by the single-center analysis and patient selection. As the different subgroups of variant histologies are rare, the study cohort, especially for ADENO and SARCO, is small and might limit conclusions when looking at subgroup analysis regarding the prognostic value of Nectin-4 in this cohort. However, we present one of the largest single-center cohorts with a long, structured follow-up.

## 5. Conclusions

Variant histologies of BC present at advanced tumor stage at the time of RC. Nectin-4 is expressed at higher levels in SCC and ADENO compared to SARCO. In our cohort with variant histologies, the expression of Nectin-4 was not prognostic for PFS or OS. However, the high expression of Nectin-4 makes ADCs highly promising in high-risk and metastatic patients and requires prospective trials, especially for SCC and ADENO. Nectin-4 should be further investigated as a potential biomarker in prospective trials of enfortumab vedotin in variant histologies, especially if patients with SARCO are included.

## Figures and Tables

**Table 1:** Patient characteristics.

Variable	SCC (*n* = 59)	ADENO (*n* = 22)	SARCO (*n* = 24)	*p* Value
Median follow-up (months)	15	22.3	7	0.089
Range	0–175	0–93	0–121	
Median age (y)	66	69	61.5	0.497
Range	37–85	41–85	51–89	
Gender				
Male	30	15	11	0.267
Female	29	7	13	
T-Stage				0.635
≤pT1	2	1	0	
pT2	8	1	1	
pT3	34	14	18	
pT4	15	6	5	
Nodal stage				0.005
N0	35	8	13	
N1	15	9	3	
N2	1	0	5	
Nx	8	5	3	
M-stage				0.466
M0	54	18	21	
M1	5	4	3	

## Data Availability

Primary data are available for bonafide researchers upon request from the corresponding author.

## Supplementary Materials

The following supporting information can be downloaded at: https://www.mdpi.com/article/10.3390/cancers14184411/s1, Figure S1: Variant histologies of bladder cancer and Nectin-4 expression levels, high magnification.
