# Supplementary material for: Expression of Nectin-4 in Variant Histologies of Bladder Cancer and Its Prognostic Value—Need for Biomarker Testing in High-Risk Patients?
Source: Cancers (Basel). 2022 Sep 11;14(18):4411. doi: 10.3390/cancers14184411 (PMC9497069; doi:10.3390/cancers14184411)
Supplement: Supplementary file 1 [file cancers-14-04411-s001.zip › cancers-1862866-supplementary.pdf]

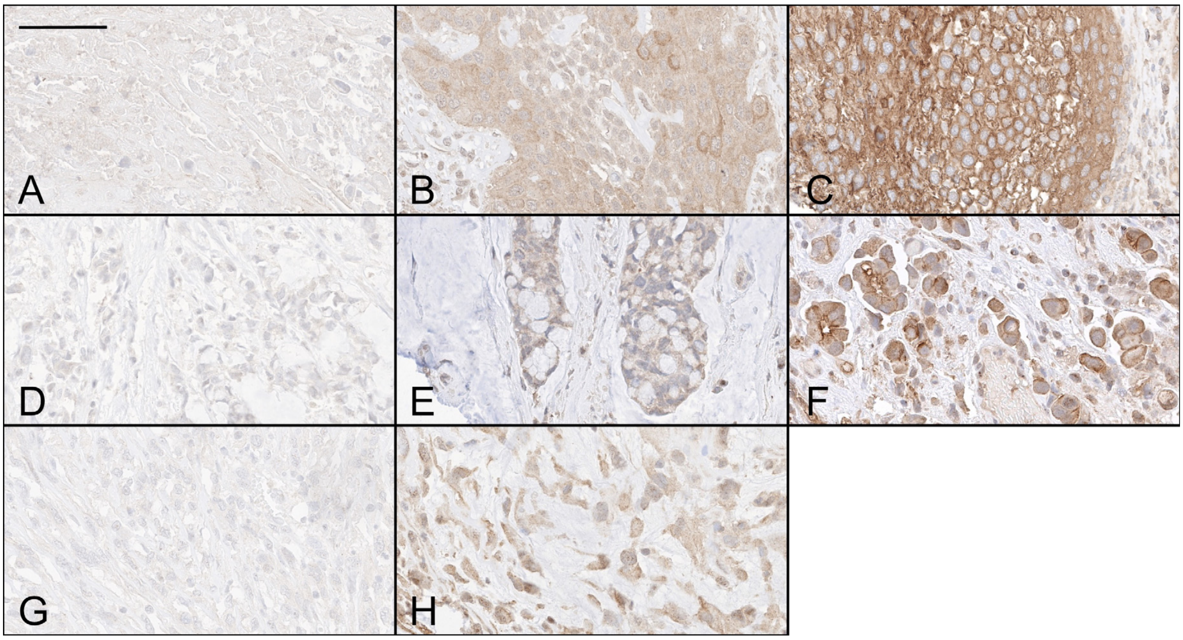

Supplementary Figure S1: Variant histologies of bladder cancer and Nectin-4 expression levels, high magnification. A–C show representative photomicrographs of pure SCC with low (A), intermediate (B), and high Nectin-4 expression (C). D–F depict representative photomicrographs of pure ADENO and weak (D), intermediate (E), and high Nectin-4 expression (F). G and H show representative photomicrographs of SARCO with weak (G) and intermediate (H) Nectin-4 expression. In SARCO, there was no high Nectin-4 expression detected. SCC, pure squamous cell carcinoma of the bladder; ADENO, adenocarcinoma of the bladder; SARCO, sarcomatoid urothelial carcinoma of the bladder; scale bar 50 µm.
